# The Dopamine Transporter Is a New Target for Ischemic Stroke

**DOI:** 10.1111/cns.70092

**Published:** 2024-10-28

**Authors:** Yan‐Qiong Cheng, Ruo‐Xi Zhang, Xing‐Yuan Li, Xiao‐Ting Zhou, Ming Chen, Ai‐Jun Liu

**Affiliations:** ^1^ Department of Pharmacy Research, Yueyang Hospital of Integrated Traditional Chinese and Western Medicine Shanghai University of Traditional Chinese Medicine Shanghai China; ^2^ MOE Frontier Center for Brain Science, Institutes of Brain Science, State Key Laboratory of Medical Neurobiology Fudan University Shanghai China

**Keywords:** D_1_ receptor, dopamine, dopamine transporter, ischemic stroke, miniature excitatory postsynaptic current, miniature inhibitory postsynaptic current

## Abstract

**Aims:**

Dopamine transporter (DAT) can regulate DA homeostasis and has been implicated in many nervous system diseases. Whether DAT is involved in the protection against ischemic stroke is unclear.

**Methods:**

In vivo microdialysis measurements of DA were recorded in the ischemic penumbral area of mice with middle cerebral artery occlusion (MCAO). DAT coding gene, *Slc6a3* mutation, and DAT overexpression animals were performed MCAO. Madopar (compound formulation of levodopa) and nomifensine (DA reuptake inhibitor) were administered in MCAO animals. Brain slices were prepared in *Slc6a3* mutation or wild‐type (WT) animals with MCAO to record miniature excitatory postsynaptic currents (mEPSCs) and miniature inhibitory postsynaptic currents (mIPSCs). The effects of DA and its dopamine‐1 receptor (D_1_R) antagonists (SCH‐23390) on mEPSCs, mIPSCs, and neurons protection were recorded.

**Results:**

MCAO caused a prominent increase in DA. *Slc6a3* mutation significantly attenuated the ischemic injury, whereas DAT overexpression aggravated this injury. Both nomifensine and madopar protected against brain injury. *Slc6a*3 mutation and DA restored the disturbance of mEPSCs and mIPSC, and protected against neuron death, which was abolished by SCH‐23390.

**Conclusion:**

DAT inhibition might be explored as a strategy for ischemic stroke prevention. DA and D_1_R involve in the restoration of synaptic dysfunction and neuron protection.

## Graphical abstract

Dopamine transporter (DAT) inhibition or high level of DA treatment could be explored as a new strategy for ischemic stroke. DA and its D_1_R might be an important mechanism for the restoration of the balance of synaptic function and neurons protection against ischemic stroke.

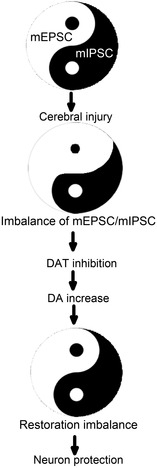

**Abbreviations**
ACh: acetylcholine
ACSF: artificial cerebrospinal fluid
ADHD: attention deficit hyperactivity disorder
ANOVA: analysis of variance
CBF: cerebral blood flow
CCK8: Cell Counting Kit‐8
CMC: carboxy methyl cellulose sodium
D_1_R: Dopamine‐1 receptor
DA: dopamine
DAT: dopamine transporter
DMEM: Dulbecco's Modified Eagle Medium
E/I: excitatory and inhibitory
GABA: γ‐aminobutyric acid
i.c.v.: intracerebroventricular
i.g.: intragastric
LV‐GFP: lentiviral vector encoding green fluorescent protein
LV‐*Slc6a3*
: lentiviral vector encoding *Slc6a3*
MCA: middle cerebral artery
MCAO: middle cerebral artery occlusion
MDD: major depressive disorder
mEPSCs: miniature excitatory postsynaptic currents
mIPSCs: miniature inhibitory postsynaptic currents
OGD: oxygen–glucose deprivation
PD: Parkinson's disease
SD rats: Sprague–Dawley rats
SUDs: substance use disorders
TTC: 2,3,5‐triphenyltetrazolium chloride
WT: wild type

## 1 Introduction

The dopamine transporter (DAT) is a transmembrane sodium chloride‐dependent protein selectively expressed in dopaminergic cells. DAT is one of the principal regulators of synaptic DA transmission [1, 2]. The DAT coding gene, *Slc6a3* in the human chromosome five, mainly affects the function and density of DAT and then alters DA reuptake activity and the dynamics of DA neurotransmission [3]. DA plays a crucial role in multiple central nervous functions and is implicated in cardiovascular diseases and cancers [4, 5, 6].

DAT also contributes to pathophysiology in both central and peripheral nervous systems, especially in central nervous system diseases, such as Parkinson's disease (PD), substance use disorders (SUDs), major depressive disorder (MDD), and attention deficit hyperactivity disorder (ADHD). It might be a main target of antipsychotic drugs and involve in the treatment to reverse antipsychotic treatment failure [7, 8].

For stroke study, cerebral ischemic injury caused hyperactive behavior associated with increased DA concentration, normalization of DA neuron density, and decreased DAT expression [9]. A recent report also showed that ischemic injury in newborn rats induced mood disorders, which was associated with alterations in the DA system [10]. Whether DAT is involved in the protection against ischemic stroke has not been fully understood. Furthermore, the possible substrates of DAT have not been fully defined. Indeed, a clear, direct relationship between DA and ischemic stroke has been controversial so far [11]. In this study, we used DAT gene mutation mice and overexpression method to show that the inhibition of DAT might be a new strategy for the treatment of ischemic stroke.

## 2 Methods

### 2.1 Animals

Homozygous (*Slc6a3* ^*tm1.1* (*cre*) *Bkmn*^) mutation and heterozygous mice were purchased from the Jackson Laboratory (stock number 006660). We identified the gene type in accordance with the instructions provided by the company. Male C57BL/6J mice and Male Sprague–Dawley (SD) rats were purchased from Sino‐British SIPPR/BK Lab Animal Ltd. (Shanghai, China).

The animals were housed at a controlled temperature (23°C–25°C) and lighting (8:00 a.m.–8:00 p.m. light, 8:00 p.m.–8:00 a.m. dark), and they had free access to food and tap water. All the animals used in this study received humane care in compliance with Yueyang Hospital of Integrated Traditional Chinese and Western Medicine, Shanghai University of Traditional Chinese Medicine for health and care of experimental animals (Abbreviated as Yueyang Hospital). And all experiments were approved by Yueyang Hospital.

### 2.2 Middle Cerebral Artery Occlusion (MCAO)

Male mice or SD rats were subjected to transient ischemia (1 h) via MCAO as described previously [12]. More details are shown in the Data S1.

### 2.3 Microdialysis and Neurotransmitters Measurement

As the ischemic penumbral area is the major target for neuroprotective drugs, monitoring metabolic parameters by microdialysis in the hippocampus in the ischemic penumbral area will be a useful tool to investigate effectiveness and mechanisms of potential target [13]. Dialysis samples were assayed for DA by using high‐performance liquid chromatography—electrochemistry (HPLC‐EC, BAS PM‐92E/LC‐4C; USA) as described with some modifications [14]. Measurements of glutamate concentrations in the dialysis samples were performed by HPLC with the use of derivatization and fluorescence detection, essentially as previously described [15, 16, 17]. More details are shown in the Data S1.

### 2.4 Brain Slice Preparation and Whole‐Cell Voltage‐Clamp Recording

Brain slices were prepared from animals at different stages (0, 6, 24 and 72 h) after ischemic injury. Miniature excitatory postsynaptic currents (mEPSCs) and miniature inhibitory postsynaptic currents (mIPSCs) recordings were performed in penumbra area (which can be clearly defined by microscope) in hippocampus at room temperature (24°C). More details are shown in the Data S1.

### 2.5 Neurons Culture

Primary neuronal cells were obtained from the cerebral cortex of neonatal SD rats within 24 h after birth, as described previously [12]. Neurons injury was induced by oxygen–glucose deprivation (OGD). Cell survival and death were examined accordingly [12]. More details are shown in the Data S1.

### 2.6 Lentivirus Administrations

For lentivirus injection, the lentiviral vectors (1–2 μL/site; 2 × 10^6^ transduction units [TU]/site) were injected into the cortex and hippocampus of SD rats at four sites by microliter syringes (Hamilton CO, Reno, NV, USA) as described [12].

Other methods, including construction and production of lentiviral vectors, immunoblotting, and PCR, are shown in the Data S1.

### 2.7 Statistical Analysis

The animals were randomly assigned by using the random permutations table. The animal cage location was exchanged every week. The order of treatment and measurement was alternated between different groups. The investigators were blinded to the procedures when they assessed the infarct size and neurological deficit score of MCAO animals, and recorded the positive cells, etc. Results were plotted with the software GraphPad Prism 7.00 (GraphPad Software; San Diego, CA, USA). Data are expressed as the mean ± standard deviation. Shapiro–Wilk test is used to assess data normality distribution. When the normality test of variance is passed, data are analyzed with a two‐tailed Student's *t*‐test or one‐way or two‐way analysis of variance (ANOVA). Data that do not exhibit a normal distribution are analyzed via a non‐parametric equivalent. *p* < 0.05 is considered statistically significant.

## 3 Results

### 3.1 The Acute Pathophysiological Changes of DA and Glutamate in MCAO Mice

#### 3.1.1 Elevation of DA and Glutamate Levels in MCAO Mice

**FIGURE 1:**
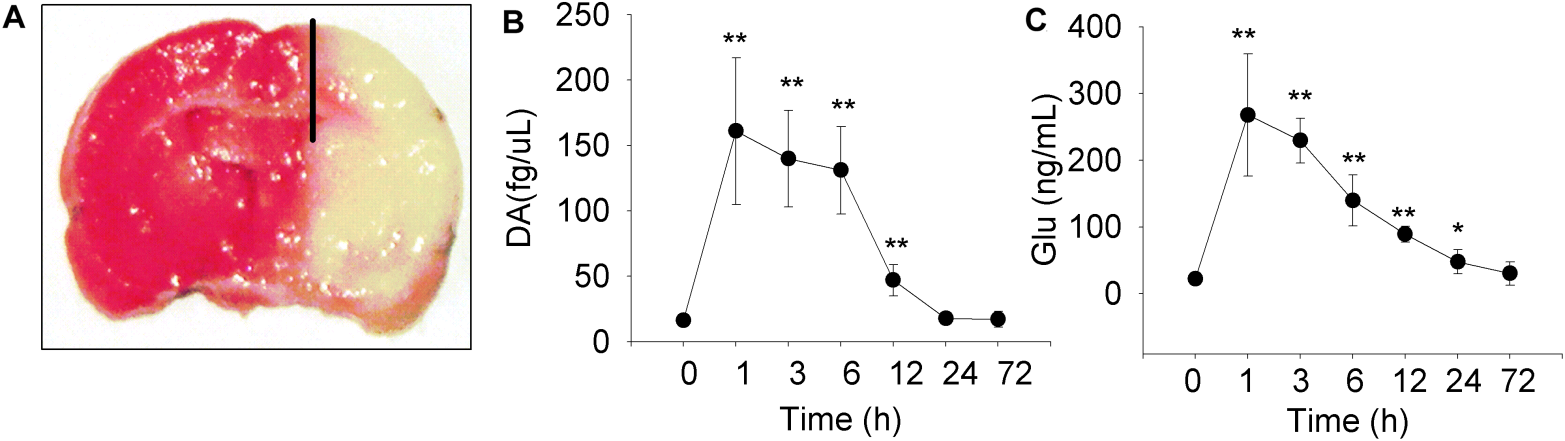
Microdialysis measurement of dopamine (DA) and glutamate (Glu) in the ischemic penumbra area of mice with MCAO. The figure shows the change in extracellular concentrations of three transmitters at different time after MCAO (*n* = 6). (A) Location of the probe was in the ischemic penumbra area of hippocampus as indicated. Brain slices were stained with 1% TTC. (B–C) The change in DA and Glu before and after MCAO. Data are shown as mean ± SD. Data are analyzed by ANOVA followed by Dunnett testing. *p < 0.05, **p < 0.01 versus before MCAO (0 h).

To define the changes of important extracellular neurotransmitters during ischemic injury, male C57BL/6J mice (20 g ±, *n* = 6 in each group) were subjected to MCAO. The extracellular levels of DA and glutamate in the ischemic penumbral area of hippocampus (Figure 1A) were detected by the microdialysis method in freely moving mice. One animal died during operation and its data were excluded.

The extracellular concentration of DA was 16.3 ± 4.16 fg/μL before operation (defined as 0 h). MCAO caused a very prominent increase in DA by about 10‐fold in the first hour after MCAO. DA remained at higher level at 6 h by about eightfold. DA was also strongly increased at 12 h after MCAO by about threefold and recovered after 24 h to baseline levels (levels before operation) (Figure 1B).

The glutamate level was significantly increased by more than 10‐fold in the first hour after MCAO. Then the concentration of glutamate gradually decreased, and remained at higher level at 24 h by about twofold. The concentration recovered at 72 h (Figure 1C).

### 3.2 The Extracellular Concentration of DA Is High Both in Normal and Cerebral Ischemic Homozygous *Slc6a3* Mutation Mice

**FIGURE 2:**
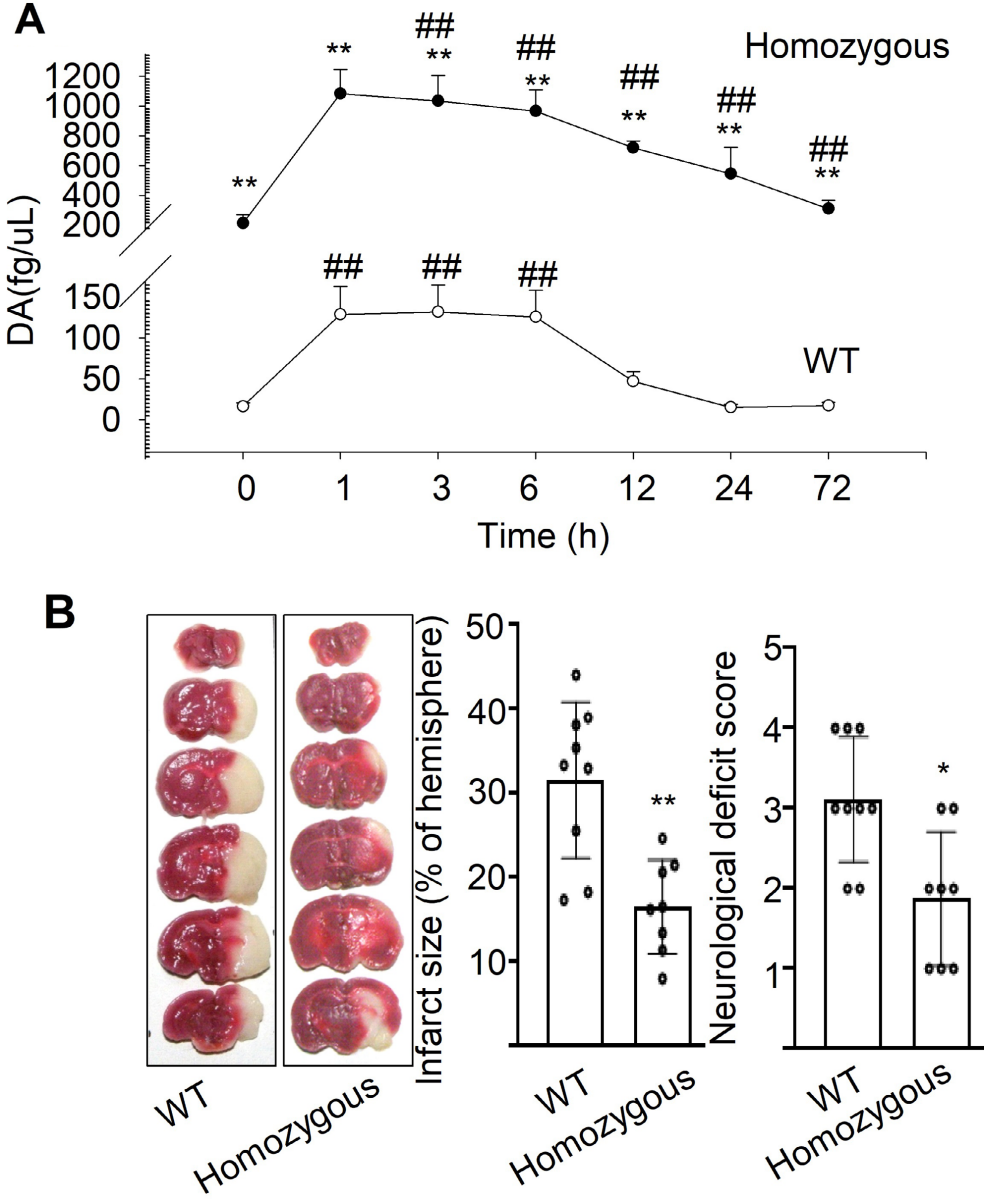
*Slc6a3* mutation increases the extracellular concentration of DA and protects against ischemic cerebral injury. (A) Microdialysis measurement to show the changes of DA levels in ischemic penumbra area of hippocampus of *Slc6a3* homozygous mutation and WT mice before and after MCAO (*n* = 5). (B) The left panel shows representative 1% TTC staining images of coronal brain sections of mice 24 h after MCAO (*n* = 10). The middle panel shows the infarct area and the right panel shows the neurological deficit scores. Data are shown as mean ± SD, and analyzed by student's *t‐*test in (B) for infarct size or non‐parametric Kruskal–Wallis test for neurological deficit scores, and two‐way ANOVA in (A). *p < 0.05, **p < 0.01 versus WT. #*p* < 0.05, ##*p* < 0.01 versus before MCAO (0 h).

DAT is one of the principal regulators of DA [2]. To investigate the effect of DAT on the regulation of DA and ischemic stroke, male *Slc6a3* homozygous mutation and WT mice were subjected to MCAO. We measured DA level in the ischemic penumbral hippocampus before MCAO (defined as 0 h) and different time after operation (*n* = 5, 20 g ±). The extracellular concentration of DA was significantly higher in *Slc6a3* mutation mice before MCAO (214 ± 56.6 vs. 16.5 ± 4.36 fg/μL in WT mice, *p* < 0.01). After MCAO, the concentration of DA in WT mice was significantly increased by about eightfold and remained at higher level at 6 h. DA was recovered to baseline levels at 72 h (Figure 2A). In *Slc6a3* mutation mice, DA was significantly increased by about fourfold and remained at higher level. *Slc6a3* mutation caused a significant increase in DA both before and after MCAO (Figure 2A).

### 3.3 *Slc6a3* Mutation Protects Against Ischemic Cerebral Injury

The effect of DAT on ischemic stroke was investigated by using *Slc6a3* homozygous mutation and WT mice (*n* = 10, 20 g ±). After MCAO, one animal in mutation group was discarded because cerebral blood flow (CBF) reduction was < 70%. As shown in Figure 2B, *Slc6a3* mutation reduced the infarct size (16.6% ± 5.99% vs. 31.6% ± 11.1% in control, *p* < 0.01), and improved neurological function (1.9 ± 0.8 vs. 3.0 ± 0.8, *p* < 0.05).

### 3.4 Nomifensine and Madopar Protect Against Ischemic Cerebral Injury

**FIGURE 3:**
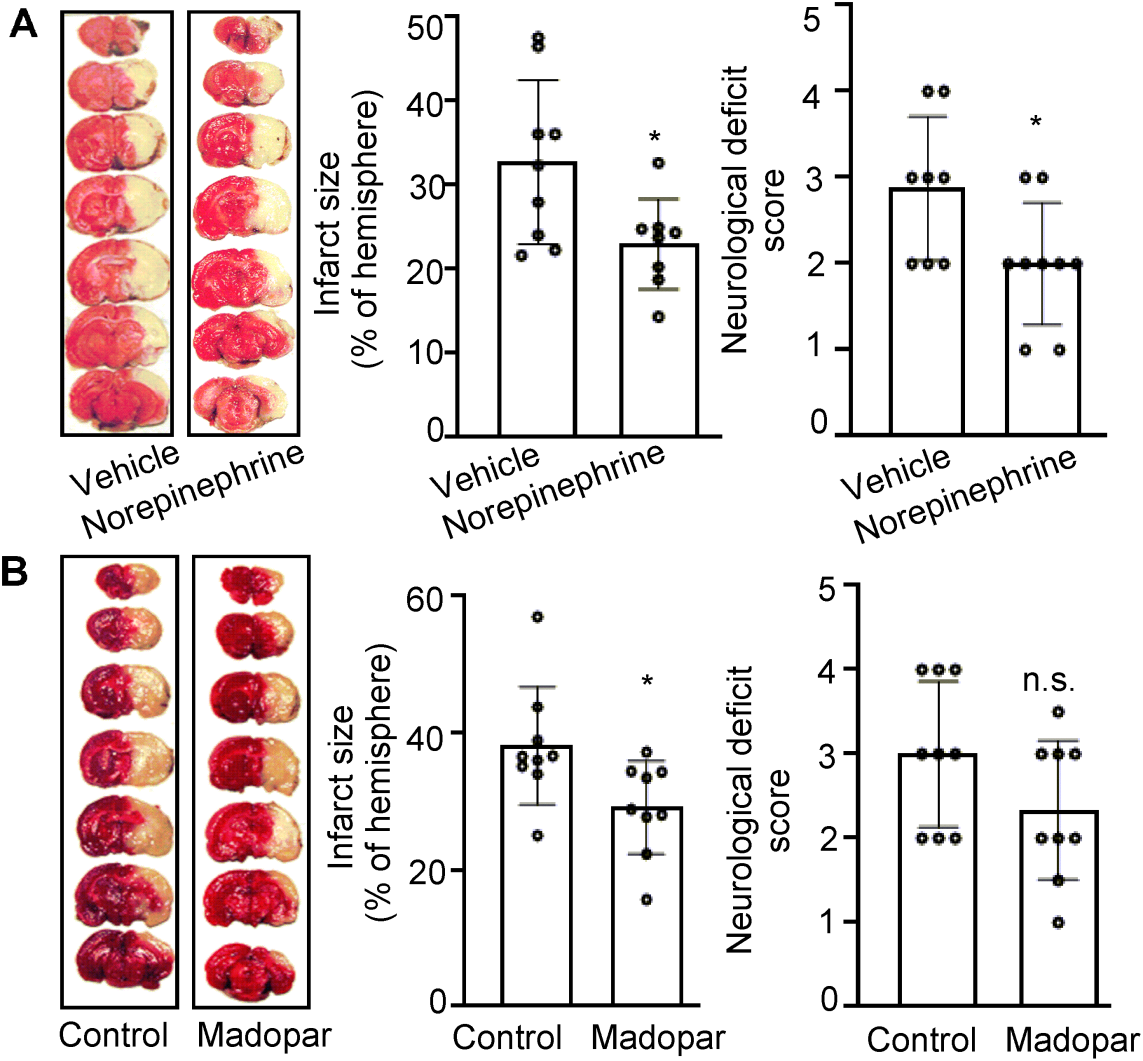
Nomifensine and madopar reduced the infarct size and improved neurological function in rats with MCAO. (A) and (B) The left panel shows representative 1% TTC staining images of coronal brain sections of rats 24 h after MCAO. The middle panel shows the infarct area and the right panel shows the neurological deficit scores. (A) Rats were administered vehicle (10 μL, i.c.v. *n* = 10) or nomifensine (100 μM, 10 μL, i.c.v. *n* = 10). (B) Rats were administered vehicle (0.5% CMC, i.g. *n* = 10) or madopar (20 mg/kg, i.g. *n* = 10). Data are shown as mean ± SD and analyzed by student's *t‐*test or non‐parametric Kruskal–Wallis test for neurological deficit scores. *p < 0.05 versus vehicle group. n.s, no significance.

We designed a series of experiments to further investigate whether DAT or DA is involved in the protective effect against stroke. Nomifensine and madopar were used in the following study. Nomifensine is a catecholamine reuptake inhibitor, and can decrease the amount of DA depletion, which involves in the function of DAT [18, 19]. Madopar is a compound formulation composed of levodopa and decarboxylase inhibitor benzylhydrazine in a ratio of 4:1. It can increase the level of DA in the brain with the presence of decarboxylase [20]. Firstly, 20 male SD rats (220 g ±) were randomized, divided into two groups, and administered vehicle (artificial cerebrospinal fluid [ACSF] 10 μL, intracerebroventricular injection [i.c.v.]) or nomifensine (100 μM, 10 μL, i.c.v.) immediately after MCAO. Three animals were discarded because death (control) or low CBF reduction (nomifensine group). As shown in Figure 3A, nomifensine decreased the infarct size by 30%, and improved neurological function (2.0 ± 0.6 vs. 2.7 ± 0.9, *p* < 0.05). Secondly, 20 male SD rats (220 g ±) were randomized, divided into two groups, and administered vehicle 0.5% carboxy methyl cellulose sodium (CMC, intragastric [i.g.] administration) or madopar (20 mg/kg, i.g.). Then, MCAO were performed. Two animals were discarded because CBF reduction was < 70% or without infarction. Madopar significantly decreased the infarct size (29.1% ± 6.82% vs. 38.1% ± 13.4% in control group, *p* < 0.05, Figure 3B). The neurological function was improved but not significantly (2.3 ± 1.0 vs. 3.0 ± 1.0, *p* = 0.18, Figure 3B).

### 3.5 Transfection of DAT Increased the Ischemic Cerebral Injury

**FIGURE 4:**
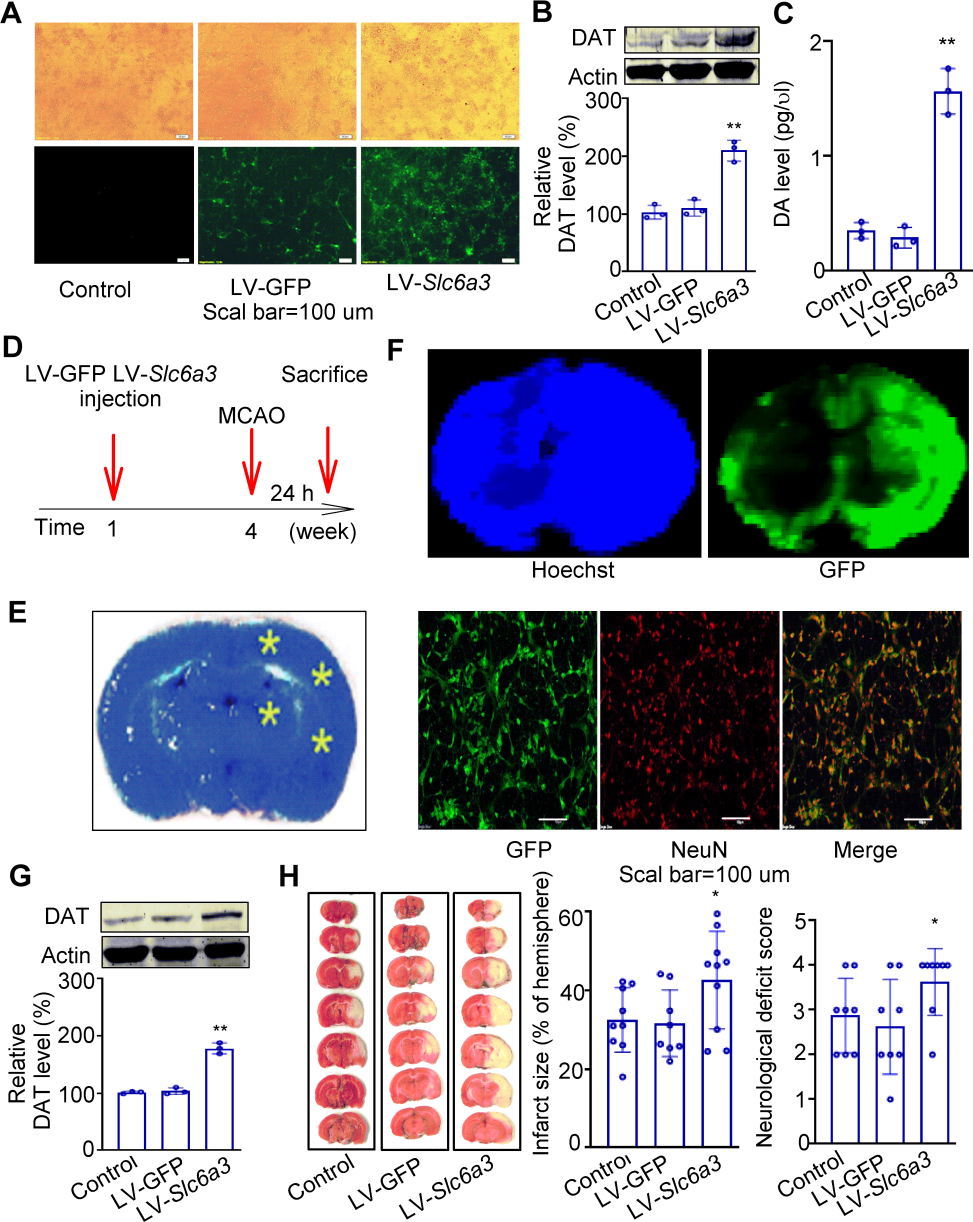
Effects of lentivirus‐mediated DAT overexpression on cerebral injury induced by MCAO. (A) Lentiviral delivery system efficiently transfected the cultured neurons. Representative image of neurons transfected with GFP‐containing lentivirus (The upper panel from light field, the lower panel from fluorescent field) for 2 days (MOI = 10). Scale bar, 100 μm. (B) Immunoblotting and quantification showing efficient overexpression of DAT in cultured cells. *n* = 3. (C) Neuron cells transfected with LV‐Slc6a3, LV‐GFP, or vehicle were pretreated by DA (20 μM) for 2 h. The intracellular concentration of DA was assayed by HPLC‐EC methods. (D) Time schedule for lentivirus injection, MCAO operation, and sacrifice. (E) Four sites for injection (yellow asterisk). Lentivirus (2 × 10^6^ TU/site) was injected into the left cortex and hippocampus at four sites. (F) Upper panel: General view of lentivirus transfection in rat brain 1 week after injection (left, the brain was stained by Hoechst; right, GFP‐containing transfection site). Lower panel: LV‐GFP efficiently transfected neurons, as detected by colocalization with the neuron‐specific marker NeuN. Scale bar, 20 μm. (G) Immunoblotting and quantification show efficient overexpression DAT in local brain tissue of rats after 3 weeks, *n* = 3. (H) The left panel shows representative 1% TTC staining of seven corresponding coronal brain sections. The middle panel shows the infarct size and the right panel shows the neurological deficit scores, *n* = 10. Data are shown as mean ± SD. Data are analyzed by ANOVA followed by Tukey *post hoc* testing or by non‐parametric Kruskal–Wallis test for neurological deficit scores. *p < 0.05, **p < 0.01 versus LV‐GFP.

The key function of DAT involved in the ischemic stroke injury was further investigated by overexpression of DAT. Firstly, neurons were transfected with lentiviral vector encoding *Slc6a3* (LV‐*Slc6a3*) to detect the transfection efficiency. The control samples were transfected with corresponding empty lentiviral vector encoding green fluorescent protein (LV‐GFP). The ratio of lentivirus titer to cell count is 100:1. The transfection efficiency was maintained over 90% determined by flow cytometry (94.7% ± 2.69%, *n* = 3), with no detectable cellular toxicity. Transfection with LV‐Slc6a3 enhanced the expression of DAT (178 ± 9.50 vs. 103 ± 5.69 in LV‐GFP group, *p* < 0.01, Figure 4B). To investigate the function of DAT transfection, Neuron cells transfected with LV‐Slc6a3, LV‐GFP, or vehicle were pretreated by DA (20 μM) for 2 h. The intracellular concentration of DA was assayed by HPLC‐EC method. Overexpression of DAT increased the DA levels by 3.8‐fold (Figure 4C).

Then, the overexpression of DAT was conducted in the brain of male SD rats (*n* = 13 in each group, three for immunohistochemistry and protein level measurement, 10 for MCAO). A time schedule for the experiment is shown in Figure 4D. LV‐Slc6a3 and LV‐GFP were stereotaxically injected into the left hemisphere (Figure 4E) according to previous report [12]. Four weeks after injection, the animals were subjected to MCAO.

The successful transfection of lentivirals was first observed by the location of GFP in the left side of brain (Figure 4F). The efficient transfection was also confirmed by immunohistochemistry with the neuron‐specific marker NeuN (the lower panel of Figure 4F). Local injection of LV‐*Slc6a3* led to approximately 72% increase in DAT protein level (*n* = 3, Figure 4G).

Then, MCAO was subjected in the other animals (*n* = 10). Overexpression of DAT significantly increased the infarct size (44.7% ± 12.4% vs. 32.5% ± 8.2% in LV‐GFP group, *p* < 0.05), and neurological deficit score (3.6 ± 0.7 vs. 2.9 ± 0.8, *p* < 0.05, Figure 4H).

### 3.6 Cerebral Ischemic Injury Destroyed the Balance Between Excitatory and Inhibitory Synaptic Function

**FIGURE 5:**
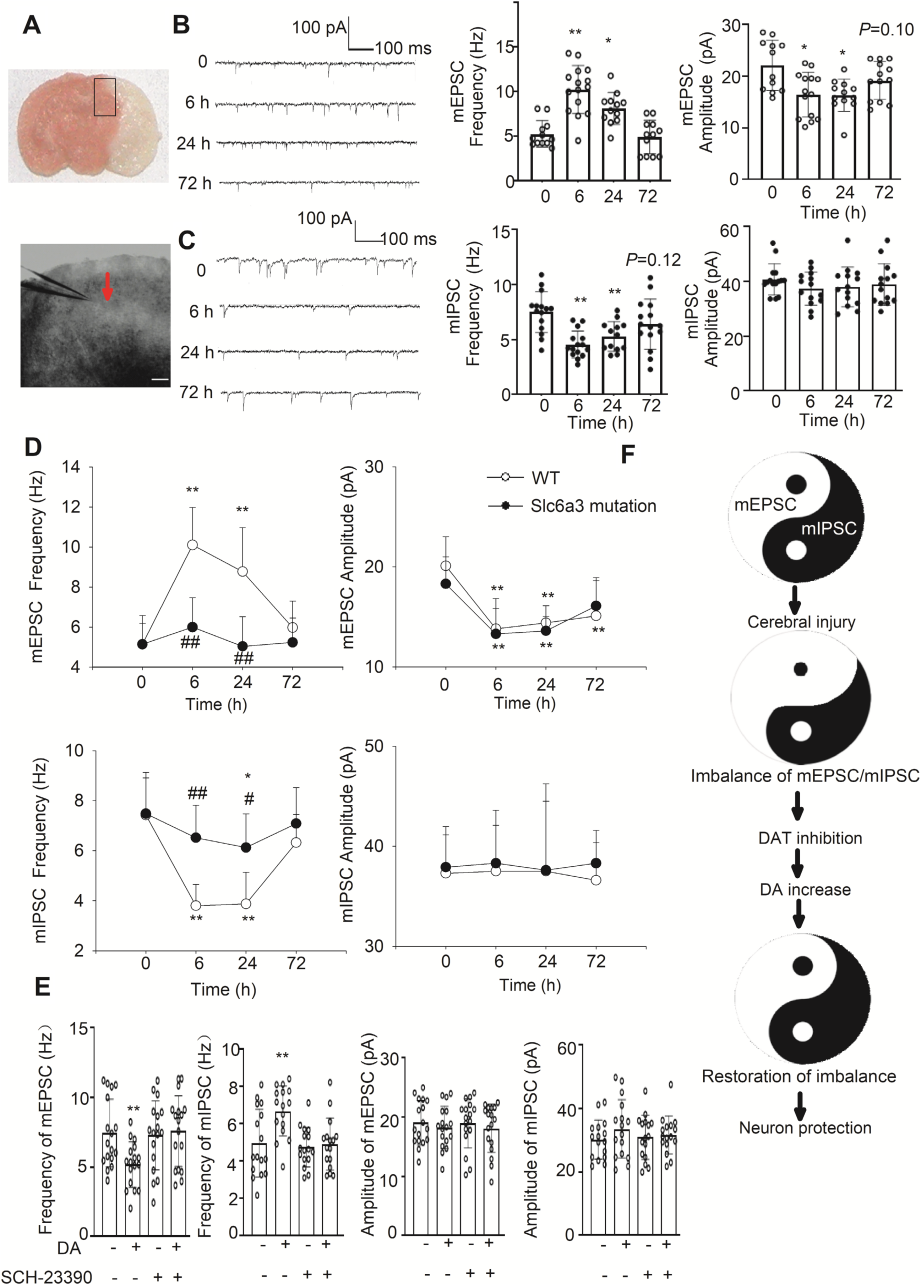
*Slc6a3* mutation and DA restore the imbalance of synaptic function induced by MCAO. (A) Bright‐field images of a recorded neuron (arrow) in ischemic penumbra area (upper panel, area in black square frame), Scale bar: 100 μm. (B, C) Traces of mEPSCs (B) and mIPSCs (C) and summary graphs showing the frequency (middle panel) and amplitude (right panel) before operation (0 h, *n* = 12 cells/4 mice both for mEPSCs and mIPSCs), 6, 24, and 72 h after MCAO (*n* = 15 cells/5 mice) in C57BL mice. (D) Summary graphs showing the frequency and amplitude before operation (0 h) and 6, 24, and 72 h after MCAO in WT and *Slc6a3* mutation mice, *n* = 15 cells/5 mice. (E) The panels show the effect of DA (1 μM), SCH‐23390 (1 μM), and their combination on the frequency and amplitude of mEPSCs and mIPSCs in the brain slices of C57BL mice 24 h after MCAO (*n* = 15 cells/5 mice in each group). (F) Proposed mechanism underlying DAT against ischemic stroke. Data are shown as mean ± SD. (B) and (C) are analyzed by one‐way ANOVA followed by Dunnet's testing. (D) by two‐way ANOVA, and (E) by one‐way ANOVA followed by Tukey testing. *p < 0.05, **p < 0.01 versus before MCAO (0 h) or vehicle. #*p* < 0.05, ##*p* < 0.01 versus WT.

The balance of excitatory and inhibitory synaptic transmission is crucial for brain function [21, 22]. The mEPSC/mIPSC frequency is often used as an indicator of excitatory and inhibitory balance [23]. To evaluate the effects of ischemic stroke injury on synapse function, we made whole‐cell patch‐clamp recording from ischemic penumbra of brain slices (Figure 5A). MCAO or sham operation was subjected in C57BL/6J mice; then, the brains were prepared in the sham group (defined as 0 h) and three MCAO groups (at 6, 24 and 72 h after MCAO, *n* = 4 in 0 h group and *n* = 5 in MCAO groups).

At 6 h after operation, the frequency of mEPSC was significantly higher in ischemic penumbra neurons (10.27 ± 2.68 Hz in 6 h vs. 5.29 ± 1.49 Hz in 0 h, *p* < 0.01; 0 h, *n* = 12 cells/4 mice; 6 h, 15 cells/5 mice) and recovered to normal level at 72. The amplitude of mEPSC was significantly lower at 6 h (16.4 ± 4.32 pA in 6 h vs. 22.1 ± 4.87 pA in 0 h, *p* < 0.01; 0 h, *n* = 12 cells/4 mice; 6 h, 15 cells/5 mice) and partly recovered at 72 h (Figure 5B).

For inhibitory synaptic function, the frequency but not the amplitude of mIPSC was significantly lower both at 6 and 24 h after MCAO (Figure 5C), indicating that ischemic injury reduced inhibitory synaptic transmission. The balance between excitatory and inhibitory synaptic transmission was significantly disturbed by ischemic injury.

### 3.7 *Slc6a3* Mutation Restores the Balance of Synaptic Function

To evaluate the effects of DAT on synapse function, we continued to make whole‐cell patch‐clamp recording in ischemic penumbra, and compared synaptic responses between *Slc6a3* mutation and WT neurons. The brain slices were prepared at 0 (sham group), 6, 24, and 72 h after MCAO (*n* = 5, 20 g ±).

For mEPSC, the results of frequency and amplitude in WT mice were similar to the data in C57BL/6J mice. The frequency was significantly higher, and the amplitude of mEPSC was significantly lower after MCAO. In *Slc6a3* mutation neurons, compared with sham operation, the frequency of mEPSC was not significantly changed after MCAO. Compared with WT mice, the frequency was significantly lower both at 6 and 24 h after operation in mutation mice. The amplitude of mEPSC was significantly lower and recovered at 72 h both in WT and mutation mice (Figure 5D, *n* = 15 cells/5 mice).

For mIPSC, similar to the data from C57BL/6J mice, the frequency but not the amplitude of mIPSC was significantly lower both at 6 and 24 h after MCAO in WT mice. *Slc6a3* mutation significantly alleviated the reduction in mIPSC frequency. For amplitude of mIPSC, there was no difference between WT and *Slc6a3* mutation mice (Figure 5D, *n* = 15 cells/5 mice). All these data indicated that Slc6a3 mutation can restore the balance of synaptic function (Figure 5F).

### 3.8 DA Restores the Imbalance of Synaptic Function Induced by Cerebral Ischemia Through the Dopamine 1 Receptor (D_1_R)

To evaluate the effects of DA on synapse function, whole‐cell patch‐clamp recording was performed in ischemic penumbra of brain slice pretreated by DA (1 μM). Because D_1_R involves in the neuroprotection against ischemia and in the depression of EPSC [24], D_1_R antagonist SCH‐23390 (1 μM) was used according to the reported reference [24].

Fifteen male C57BL/6J mice (20 g ±) were randomly divided into three groups (vehicle control, DA, and DA + D_1_R antagonist SCH‐23390 treatment group). The brain slices were prepared at 24 h after MCAO. The frequency of mEPSC was significantly lower in DA group (5.18 ± 1.63 vs. 7.45 ± 2.44 Hz in vehicle control group, *p* < 0.01, 15 cells/5 mice). At the same time, the frequency of mIPSC was significantly higher in DA group (6.70 ± 1.35 vs. 4.97 ± 1.82 pA in control group, *p* < 0.01, 15 cells/5 mice). The reduction in frequency of mEPSC and the increase in mIPSC were completely abolished by D_1_R antagonist SCH‐23390 (Figure 5E). For amplitude of mEPSC and mIPSC, there was no difference between four different groups (Figure 5E). All these data indicated that DA restored the imbalance of synaptic function induced by ischemic injury. This function might be mediated by D_1_R.

### 3.9 The Protection of DA Against Neuron Death Is Related to D_1_R

**FIGURE 6:**
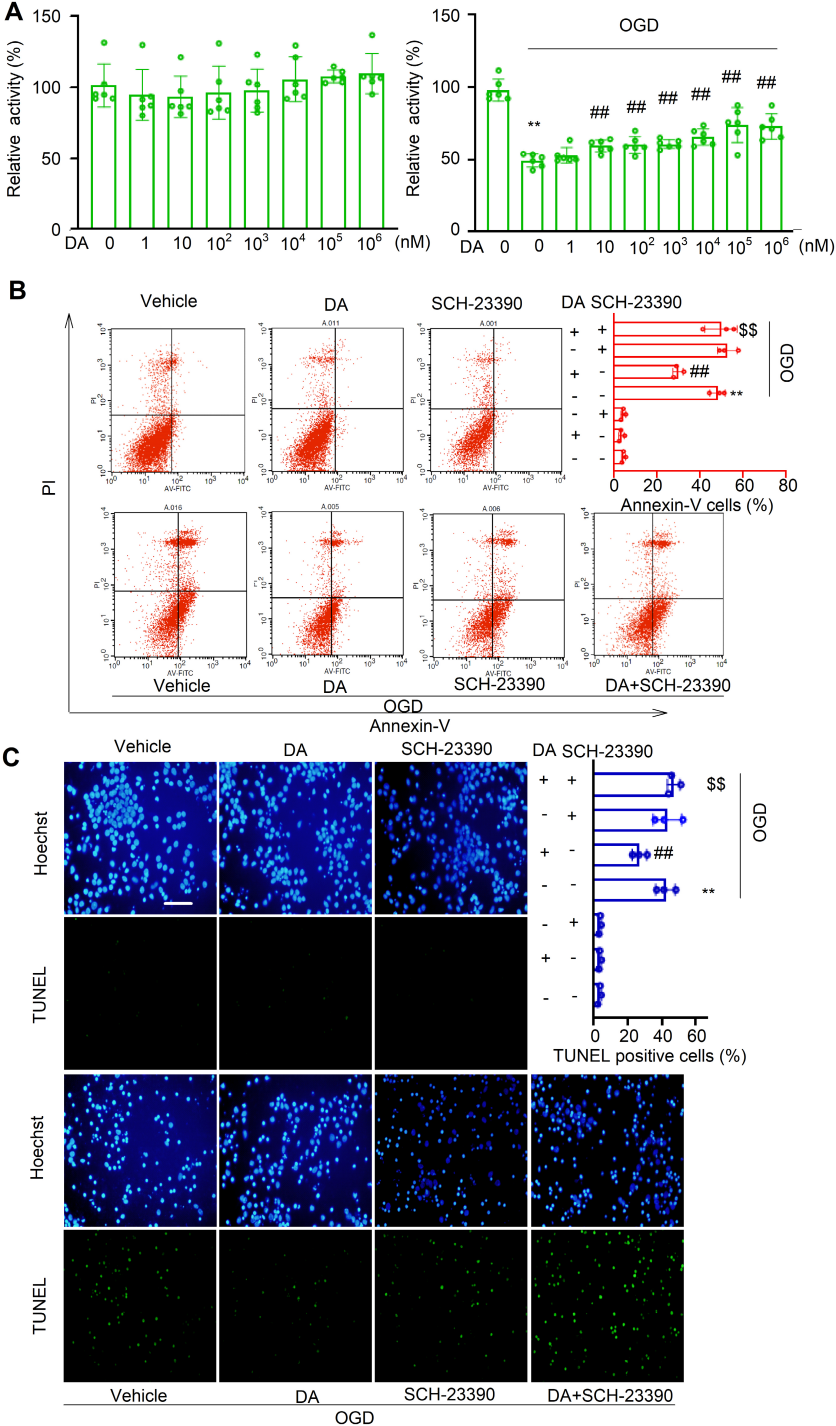
Protection of DA against neuron death is related to D_1_R. (A) In cultured primary neurons, the cells were treated by DA with (left) or without (right) OGD. The cell number and cell viability were assessed by CCK8. (B, C) The neurons were treated by DA (0.1 μM) or D_1_R antagonist SCH‐23390 (0.1 μM) under OGD for 12 h. The cell death (Annexin V staining and PI staining) was analyzed by flow cytometer (B) or by TUNEL staining kit and Hoechst 33342 (C) Scale bar, 20 μm. *n* = 3 in each group. Data are analyzed by one‐way ANOVA followed by Tukey testing. **p < 0.01 versus normal vehicle control. #*p* < 0.05, ##*p* < 0.01 versus vehicle group exposed in OGD. $$*p* < 0.01 versus DA group exposed in OGD.

In cultured primary neurons, the cell number and cell viability were not affected by DA within the certain dosage range (0–10^6^ nM). DA significantly increased the cell viability induced by OGD (10–10^6^ nM, *p* < 0.01) (Figure 6A).

We further investigated the protective effect of DA against ischemic injury in cultured primary neurons. As assessed by flow cytometric analysis of annexin V staining, the neuron death induced by OGD was significantly decreased by DA (1 μM) (29.7% ± 2.45% vs. 48.4% ± 3.56% in OGD group, *p* < 0.01). The reduction in neuron death by DA was abolished by the D_1_R antagonist SCH‐23390 (1 μM) (Figure 6B). Similar results were obtained by TUNEL analysis (Figure 6C). All these data indicated that the protection of DA against neuron death might be mediated by D_1_R.

## 4 Discussion

DAT regulates DA homeostasis by transporting extracellular DA into the intracellular space that controls the synaptic levels of DA. It is implicated in a wide range of physiological and pathological functions. For example, DAT might be an important mechanism of PD [25], and the DAT Scan has been an important imaging technique for the diagnosis of PD [26]. DAT inhibitors theoretically represent an attractive way to alleviate parkinsonism [27]. At the same time, DAT has been correlated to many environment‐sensitive psychiatric diseases including MDD, ADHD [7, 8].

Different from the situation in the PD and psychotic diseases, the studies in the brain ischemic injury are largely missing. This might partly correlate to the neurotransmitter, DA, which is regulated by DAT. Indeed, the function of DA on ischemic stroke has been controversial. Globus et al. reported that microdialysis studies demonstrated that ischemia induced an 500‐fold increase in DA concentrations in the neostriatum [28]. This group also showed the degree of ischemic neuronal damage was markedly attenuated by DA depletion in the striatum of rats with cerebral ischemia [11].

On the other side, delayed treatment with levodopa significantly contributes to the recovery of neurological function after MCAO in rats [29]. Another report showed that systemic administration of a D_1_R agonist confers neuroprotection against ischemia [30]. Dopamine D_2_ receptor agonists also have been concluded as a neuroprotection against ischemic injury [31].

Our data show that the inhibition of DAT by nomifensine can protect against ischemic injury. As a catecholamine reuptake inhibitor, nomifensine potently inhibits the reuptake of norepinephrine and DA, which might interfere with other pathways [18, 19]. Accordingly, mutation of DAT is also used in the following experiments and these data both conclude the protective effect of DAT inhibition. At the same time, pretreated by madopar is also beneficial for ischemic stroke.

Our findings also suggest that the restoration of balance between excitatory and inhibitory synaptic function, and the protection of DA—D_1_R pathway against neuron death play a critical role in the mechanism of DAT inhibition.

The balance of major excitatory and inhibitory is named as excitatory and inhibitory (E/I). As the two most important neurotransmitters in the central nervous system, glutamate and γ‐aminobutyric acid (GABA) mediate E/I neurotransmission, respectively [32, 33], and the balance of E/I synaptic transmission is critical for proper information processing [34]. Usually, mEPSC/mIPSC frequency is often used as an indicator of E/I balance [23], especially recorded in isolated brain slices [35].

Reports have shown that the disturbance of E/I balance underlay many neuropsychiatric illnesses, including autism spectrum disorder and schizophrenia, as well as anxiety. The disturbance of E/I balance during mental processing, enhanced excitatory transmission, or reduced inhibitory transmission resulted in anxiety‐like behaviors [36, 37].

For ischemic stroke, the balance in the glutamate level is disturbed by ischemic injury, and the extracellular glutamate concentration increases in an uncontrollable manner. A large accumulation of glutamate induces the cellular overload of calcium and the release of free radicals. Neuronal cells die inevitably. All these aggravate the cerebral ischemic injury [32, 38, 39].

Consistent with these previous data, our results also showed the significantly increase in glutamate in the brain during the ischemic injury, especially the first 24 h after MCAO. The E/I balance was markedly disturbed, the frequency of mEPSC was significantly increased, whereas the mIPSC was decreased.

Our results also identified the inhibition of DAT is a critical regulator to restore the balance of synaptic function (Figure 5F). High level of DA might be an important mechanism for the restoration of synaptic imbalance.

Indeed, ischemia induced a great increase in DA in certain area of brain [14, 28]. We also got the similar result in the ischemic penumbra of MCAO animals. Our data suggest that high level of DA induced by ischemic injury might only be a result but not a cause aggravating cerebral injury according to the following reasons: First, compared with WT mice, DAT (*Slc6a3*) mutation significantly increased DA level more than 10‐fold, while incredibly attenuated the ischemic injury. Second, madopar can increase the level of DA in brain, and protects against ischemic injury. Third, DA can restore the balance of synaptic function and reduce the neuron death. So DA is not a cause of cerebral ischemia. Conversely, it might be an important protective factor against ischemic stroke.

Previous reports show that the subtype of DA receptor, D_1_R, can inhibit synaptic glutamate release after ischemia and might underlie the neuroprotective mechanisms of D_1_R activation. D_1_R activation is also involved in the correlation between mEPSC depression and alleviated cell death [24, 30]. Our study also identified the D_1_R mediated the protective effect of DA on the restoration of synaptic dysfunction induced by ischemic injury.

## 5 Conclusion

DAT inhibition or high level of DA treatment could be explored as a new strategy for ischemic stroke. DA and its D_1_R might be an important mechanism for the restoration of the balance of synaptic function and neuron prevention against ischemic stroke.

## Author Contributions

Ai‐Jun Liu conceived the idea. Ai‐Jun Liu and Yan‐Qiong Cheng designed the experiments. Yan‐Qiong Cheng, Ruo‐Xi Zhang, and Xiao‐Ting Zhou performed the experiments for animal experiments. Xing‐Yuan Li contributed to the immunoblotting and PCR. Ruo‐Xi Zhang contributed to the detection of neurotransmitters. Ming Chen contributed to the whole‐Cell Voltage‐Clamp Recording. Yan‐Qiong Cheng and Ruo‐Xi Zhang analyzed the data. Ai‐Jun Liu and Yan‐Qiong Cheng wrote the paper.

## Conflicts of Interest

The authors declare no conflicts of interest.

## Supporting information


Data S1.

## Data Availability

The data that support the findings of this study are available from the corresponding authors.
