# Supplementary material for: The Dopamine Transporter Is a New Target for Ischemic Stroke
Source: CNS Neurosci Ther. 2024 Oct 28;30(10):e70092. doi: 10.1111/cns.70092 (PMC11518691; doi:10.1111/cns.70092)
Supplement: Supplementary file 1 — Data S1. [file CNS-30-e70092-s001.docx]

**Supplementary information**

**Methods**

**Middle Cerebral Artery Occlusion (MCAO)**

Male mice or SD rats were subjected to transient ischemia (1 h) via MCAO as described previously^1^. Animals were anesthetized with 3.0% isoflurane and inhaled 1%-2.0% isoflurane in air spontaneously by use of a mask. Core body temperature (rectum) was maintained at 37℃ with a heating pad (Nanjing Xin Xiao Yuan Biotech, Nanjing, China) during surgery. The left MCAO surgery in animals was subjected. Nylon filaments were used for MCAO (for mice 20 g ±, length 33 cm, diameter 0.14±0.01 mm and top diameter 0.20±0.01 mm (top length 6 mm); for rats 220 g ±, length 45 cm, diameter 0.22±0.01 mm and top diameter 0.32±0.02 mm (top length 6 mm) (Beijing cinontech co. Ltd, Beijing China). A nylon filament was inserted into the external carotid artery and gently advanced through the internal carotid artery until its tip occluded the origin of the MCA, and external carotid artery was occluded permanently. The change of cerebral blood flow (CBF) supplied by the left middle cerebral artery (MCA) was confirmed by a laser Doppler transducer (MNP110XP, ADInstruments, [Australia](http://www.iciba.com/Australia)) to a laser Doppler computerized main unit (ML191, ADInstruments, [Australia](http://www.iciba.com/Australia)). Animals that did not show a CBF reduction of at least 70% were excluded from the experimental group, as were animals that died after ischemia induction. One hour after MCAO, the occluding filament was withdrawn to allow reperfusion. Twenty-four hours after surgery, neurological deficit scores of 5 points were recorded. Then animals were anesthetized and killed by high dose of sodium pentobarbital (10%, 2.0 mL/kg, intraperitoneal injection) for various examinations after neurological evaluation.

The neurological deficit scores of 5 points: The scores were measured after the MCAO procedure. The following scoring criteria were employed: 0 points: no observable deficit; 1 point: forelimb flexion, with mild neurological impairment; 2 points: unidirectional circling, with moderate neurological impairment; 3 points: falling to the hemiplegia side, with severe neurological impairment; 4 points: inability to walk spontaneously or lack of consciousness.

**The staining of 2, 3, 5-triphenyltetrazolium chloride (TTC)**^1^ **.**

The coronal slices of brains were prepared with brain-cutting matrix (ASI Instruments, Warren, MI, USA). The slices were incubated in 1% TTC solution (Sinopharm Chemical Reagent Co. Ltd., Shanghai, China) and photographed. The possible interference of a brain edema in assessing the infarct size was corrected with a standard method of subtracting the volume of the nonischemic ipsilateral hemisphere from that of the contralateral hemisphere. The infarct size was quantified with ImageJ software.

**Microdialysis**

*In vivo* microdialysis measurements of hippocampus extracellular neurotransmitter levels (DA and glutamate) in freely moving mice were performed as described previously^2^**.** The mice were placed in a stereotaxic micromanipulator (ASI Instruments, Warren, MI, USA). The guide cannula (for later microdialysis probe insertion) was implanted into the top of hippocampus (anteroposterior (AP) -2.0 mm from bregma, mediolateral (ML) -2.0 mm, dorsoventral (DV) -2.3 mm) and fixed with acrylic bone cement (The site is in the penumbra area after MCAO). After 72 h, a 1 mm-length microdialysis probe (CMA/7 microdialysis AB, Holliston, MA, USA) with an outer diameter of 0.24 mm and a molecular weight cut-off of 6 kDa was slowly inserted into the guide cannula. Microdialysis approach was applied to collect basal levels with perfusion flow rate of 1 μL/min. One day later, mice were subjected to ischemia via MCAO on the left side. Dialysis samples were collected in tubes containing 0.1 M perchloric acid at 1, 3, 6, 12, 24 and 72 h after MCAO. Keep the perfusate temperature at 37.0℃ - 38.0℃ during dialysis. Dialysis samples were stored in liquid nitrogen before detection.

**Detection of neurotransmitters**

Dialysis samples were assayed for DA by using high performance liquid chromatography - electrochemistry (HPLC-EC, BAS PM-92E/LC-4C, USA) as described with some modifications^3^. Measurements of glutamate concentrations in the dialysis samples were performed by HPLC with the use of derivatization and fluorescence detection, essentially as previously described^4-5^.

**Brain Slice Preparation and Whole-Cell Voltage-Clamp Recording**

Brain slices were prepared from animals at different stages (0, 6, 24 and 72 hours) after ischemic injury. The animals were anesthetized with 3.0% isoflurane and decapitated. The brains were quickly removed and immersed in ice-cold artificial cerebrospinal fluid (ACSF). Coronal slices of 250 μm thicknesses were cut using a vibratome (VT1200S, Leica, Nussloch, Germany) and incubated in oxygenated ACSF for 1 h at 32℃.

Recordings were performed in penumbra area (which can be clearly defined by microscope) in hippocampus at room temperature (24℃). For whole-cell recording, patch electrodes were prepared from borosilicate glass (Φ 1.50 mm, φ 0.89 mm, Vital Sense Scientific Instruments 20132139, China) using a horizontal electrode puller (Sutter P-97, USA). The resistance of the pipette filled with intracellular solution was 2-3 ΜΩ.

For miniature Excitatory Postsynaptic Currents (mEPSCs) recording, electrodes were filled with an intracellular solution containing (mM): K-gluconate 133, NaCl 8, EGTA 0.6, Mg·ATP 2, Na_3_·GTP 0.3, HEPES 10. To allow verification of the identity of recorded neurons, sodium channel blocker tetrodotoxin (TTX 0.5 μM) and picrotoxin 100 μM were included in the solution. The membrane potential was clamped at -70 mV and recording the mEPSCs for 15 min.

For miniature inhibitory postsynaptic currents (mIPSCs) recording, electrodes were filled with an intracellular solution containing (mM): KCl 130, MgCl_2_ 1.0, EGTA 5, Na_2_·ATP 5, HEPES 5. To allow verification of the identity of recorded neurons, TTX 0.5 μM and non-NMDA receptor blocker 6,7-dinitroquinoxaline-2,3-dione (DNQX, 10 μM) were included in the solution. Detection voltage and recording time were the same as above.

**Neurons culture and drug/lentivirus administration**

Primary neuronal cells were obtained from the cerebral cortex of neonatal SD rats within 24 h after birth, as described previously^1^. One day after isolation, the cultures were replenished with neurobasal medium (Invitrogen, Carlsbad, CA, USA) supplemented with 2% B27 (Invitrogen). Glial growth was suppressed by addition of uridine (10 μM). Staining for NeuN (neuron marker, Millipore) was performed to show that cultured cells contained > 90% neurons. After 7-day culture *in vitro*, the neurons were transfected with lentivirus (multiplicity of infection (MOI) = 10) for DAT overexpression. The incubation time for transfection was 2 days. In another experiment, the cells were treated with DA, SCH-23390 or vehicle for 30 min, prior to model treatment.

**Cell Viability Assay**

Cell viability was measured by the Cell Counting Kit-8 (CCK8) reagent (Dojindo, Japan). Briefly, cells were seeded in 96-well culture plates and received the indicated treatments. Then cells were incubated with 10 µL CCK8 reagent and finally the absorbance at 450 nm was measured.

**Oxygen-glucose deprivation (OGD) model and cell injury assay**^1^

Control cultured neurons were grown in Dulbecco’s Modified Eagle Medium (DMEM) containing glucose (25 mM) and incubated under normal culture conditions. To establish OGD condition, the cultured neurons were washed 3 times, cultured in DMEM with no glucose and incubated for 12 h in a hypoxic chamber (Thermo Fisher Scientific, OH, USA), and was continuously flushed with 94% N_2_ and 5% CO_2_ at 37 ºC to obtain 1% O_2_. After 7-day culture *in vitro*, cultured cells were exposed to OGD for 12 h prior to apoptosis assay. Cell survival and death was examined by manually counting the cells double-stained with Hoechst (Beyotime, Shanghai, China) and by *in situ* cell death detection kit (TUNEL staining kit, Roche, Mannheim, Germany), respectively. The cell nuclei were counterstained with Hoechst 33342 (1 μg/mL). The dead or apoptotic cells were labeled green with the TUNEL staining kit. Images were acquired under a fluorescent microscope (IX-71; Olympus, Tokyo, Japan). The death or apoptosis rate is defined as the ratio of cells labeled green vs blue. The cell death was also detected by Annexin V/PI staining analysis (Annexin V staining for viable apoptotic cells, and PI staining for death and late apoptotic cells). Flow cytometric analysis was performed with a flow cytometer (BD FACSLyric^TM^ Flow Cytometer, USA) to count 10 000 cells for each experiment.

**Construction and production of lentiviral vectors**

The rat Slc6a3 cDNAs were polymerase chain reaction (PCR) amplified and subcloned into a pUC57 vector (Invabio, Shanghai Innovation Biotechnology Co. Ltd, Shanghai, China) with EcoRV restriction sites. Analysis of lentiviral transfection efficiency was monitored by flow cytometry (BD). Fluorescence activated cell sorting (FACS) analysis was used to detect 10,000 cells for each experiment.

**Nomifensine and lentivirus administrations**

For nomifensine administration, rats were fixed in stereotaxic instrument and were injected with 10 μL of vehicle: ACSF, nomifensine (100 μM, 10 μL) into the left lateral ventricle followed by MCAO.

For lentivirus injection, the lentiviral vectors (1-2 μL/site; 2 × 10^6^ transduction units [TU]/site) were injected into the cortex and hippocampus of SD rats at 4 sites by microliter syringes (Hamilton CO, Reno, NV, USA) as described^1^. During the intra-cerebroventricular (i.c.v.) injection, lentivirus stereotaxic injection and the following MCAO, a temperature controller pad was used to maintain the core temperature (rectum) at 37 ºC.

**Immunoblotting and PCR**

The total protein from tissue/cells was prepared. Tissue/cell extract was boiled in 4× loading buffer, subjected to SDS-PAGE, and transferred onto the pure nitrocellulose blotting membranes. The membranes were incubated with anti-DAT antibody prior to incubation with secondary antibody (Rockland). The image was captured by the Odyssey infrared imaging system (Li-Cor Bioscience, Lincoln, NE, USA). The data were analyzed using ImageJ software (NIH). All immunoblotting experiments were repeated for 3 times. The mean value from 3 experiments was used to indicate the value for each animal.

*Slc6a* mutation and wild-type (WT) animals are identified by PCR. Protocol primers are listed as 5’-3’: TGG CTG TTG GTG TAA AGT GG; GGA CAG GGA CAT GGT TGA CT; CAA AAA GAC GGC AAT ATG GT

**Reference**

1. Guo JM, Liu AJ, Zang P, et al. ALDH2 protects against stroke by clearing 4-HNE. *Cell Res* 2013;23:915–930.

2. Kiewert C, Mdzinarishvili A, Hartmann J, et al. Metabolic and transmitter changes in core and penumbra after middle cerebral artery occlusion in mice. *Brain Res* 2010;1312:101–107.

3. Fan Y, Kong X, Liu K, et al. Exercise on Striatal Dopamine Level and Anxiety-Like Behavior in Male Rats after 2-VO Cerebral Ischemia. *Behav Neurol* 2022;2022:2243717.

4. Zhang MY, Hughes ZA, Kerns EH, et al. Development of a liquid chromatography/tandem mass spectrometry method for the quantitation of acetylcholine and related neurotransmitters in brain microdialysis samples. *J Pharm Biomed Anal* 2007;44:586–593.

5. Seki Y, Feustel PJ, Keller RW, et al. Inhibition of ischemia-induced glutamate release in rat striatum by dihydrokinate and an anion channel blocker. *Stroke* 1999;30:433–440.

6. Feustel PJ, Jin Y, Kimelberg HK. Volume-regulated anion channels are the predominant contributors to release of excitatory amino acids in the ischemic cortical penumbra. *Stroke* 2004;35:1164–1168.
